# The development of aperiodic and periodic resting-state power between early childhood and adulthood: New insights from optically pumped magnetometers

**DOI:** 10.1016/j.dcn.2024.101433

**Published:** 2024-08-10

**Authors:** Marlee M. Vandewouw, Julie Sato, Kristina Safar, Natalie Rhodes, Margot J. Taylor

**Affiliations:** a Department of Diagnostic & Interventional Radiology, Hospital for Sick Children, Toronto, Canada; b Program in Neurosciences & Mental Health, Hospital for Sick Children, Toronto, Canada; c Autism Research Centre, Bloorview Research Institute, Holland Bloorview Kids Rehabilitation Hospital, Toronto, Canada; d Institute of Biomedical Engineering, University of Toronto, Toronto, Canada; e Sir Peter Mansfield Imaging Centre, School of Physics and Astronomy, University of Nottingham, Nottingham, United Kingdom; f Department of Medical Imaging, University of Toronto, Toronto, Canada; g Department of Psychology, University of Toronto, Toronto, Canada

**Keywords:** Optically pumped magnetometers, Magnetoencephalography, Development, Power, Aperiodic activity, Periodic activity

## Abstract

Neurophysiological signals, comprised of both periodic (e.g., oscillatory) and aperiodic (e.g., non-oscillatory) activity, undergo complex developmental changes between childhood and adulthood. With much of the existing literature primarily focused on the periodic features of brain function, our understanding of aperiodic signals is still in its infancy. Here, we are the first to examine age-related changes in periodic (peak frequency and power) and aperiodic (slope and offset) activity using optically pumped magnetometers (OPMs), a new, wearable magnetoencephalography (MEG) technology that is particularly well-suited for studying development. We examined age-related changes in these spectral features in a sample (*N*=65) of toddlers (1–3 years), children (4–5 years), young adults (20–26 years), and adults (27–38 years). Consistent with the extant literature, we found significant age-related decreases in the aperiodic slope and offset, and changes in peak frequency and power that were frequency-specific; we are the first to show that the effect sizes of these changes also varied across brain regions. This work not only adds to the growing body of work highlighting the advantages of using OPMs, especially for studying development, but also contributes novel information regarding the variation of neurophysiological changes with age across the brain.

## Highlights

• OPM-MEG is a new MEG technology suitable for very young children.
• Resting-state OPM data were obtained in children (1–5yrs) and adults (20–38yrs).
• Changes with age in aperiodic and periodic activity were frequency and region specific.
• These data demonstrate the spatial specificity of neurophysiological development.
• They also highlight the advantages of using OPM-MEG for studying development.

## 1 Introduction

The study of the development of neurophysiological brain signals in health and disease has a long and rich history. Neural oscillations, or the periodic fluctuations in neuronal activity, have been the primary focus, with a wealth of investigations into their role in sensory and cognitive processes, and on their marked evolution across the lifespan. While most studies have used electroencephalography (EEG), there has been increasing interest in magnetoencephalography (MEG) due to its comparable temporal and superior spatial sensitivity (Baillet, 2017, Hämäläinen and Lundqvist, 2019). However, both EEG and traditional MEG systems pose several challenges for studying the development of neural oscillations (R. M. Hill et al., 2019), particularly when examining both toddlers and adults. Periodic activity is also only one aspect of neurophysiological signals, with an increasing body of work suggesting that the background activity in the brain, or the aperiodic activity, also undergoes significant developmental changes (Voytek et al., 2015) and has distinct cognitive relevance (Thuwal et al., 2021). Here, we are the first to use optically pumped magnetometers (OPMs), a new, wearable MEG technology that is more suitable for studying very young children, to characterize the changes in both periodic and aperiodic neural activity between toddlerhood and adulthood.

Periodic activity is defined by narrow peaks of power in the frequency domain, ranging from slow frequencies such as delta through to high gamma (Buzsáki and Draguhn, 2004). In contrast, aperiodic activity does not contain rhythmic oscillations, instead, it is “scale-free”, meaning it does not contain a dominant temporal scale (B. J. He, 2014). Its distribution follows the “1/f” power law – exponentially decreasing power with increasing frequency – which can be characterised by its slope and offset (Gao et al., 2017). While many studies have attempted to link periodic or oscillatory activity with a wide range of disease states over the years, reliable biomarkers have remained elusive (e.g., Parellada et al., 2023). It is suggested that the aperiodic component may be more stable and sensitive to a range of clinical conditions (Pani et al., 2022). Importantly, aperiodic activity has been proposed as a means of determining the excitatory-inhibitory (E/I) balance in the brain (Gao et al., 2017) that has been implicated in a range of developmental conditions (see (Pani et al., 2022) for a review). However, before it can be used clinically, a better understanding of aperiodic activity across development is required.

Investigations into the development of aperiodic activity are in their infancy and have predominantly relied on EEG, with limited work using MEG (e.g., Manyukhina et al., 2022). These studies have shown that the aperiodic slope and offset both decrease with age within infancy (Schaworonkow and Voytek, 2021), childhood (A. T. Hill et al., 2022a; McSweeney et al., 2023), and adolescence (McSweeney et al., 2021), as well as extending across these developmental periods (Cellier et al., 2021, Favaro et al., 2023, Tröndle et al., 2022). On the other hand, while age-related changes in neural oscillations have been well characterised (e.g., Ebersole and Pedley, 2003), traditional analyses of neural oscillations within canonical frequency bands can be confounded by aperiodic activity (Donoghue, Dominguez, et al., 2020). For example, frequently reported decreases in alpha power in aging were found to be absent when controlling for the aperiodic slope (Cesnaite et al., 2023, Merkin et al., 2023). Thus, it is important to re-examine developmental changes in periodic activity while accounting for the influence of its aperiodic counterpart. A small selection of studies has done so, finding age-related increases in aperiodic-adjusted peak frequency in alpha (Cellier et al., 2021, McSweeney et al., 2023, Tröndle et al., 2022), but not beta (A. T. Hill et al., 2022a). Findings were mixed with respect to aperiodic-adjusted alpha power (Cellier et al., 2021; A. T. Hill et al., 2022a; Tröndle et al., 2022), however, a very well-powered sample reported increases across childhood into early adulthood (Tröndle et al., 2022).

A confound with these EEG studies, however, is that brain maturation occurs synchronously with skull thickening, which would lead to greater resistance and hence reduced EEG amplitudes (Tröndle et al., 2022). Therefore, changes in the aperiodic component seen with age could be in part secondary to physical skull changes irrelevant to brain function. The skull and scalp also limit the spatial resolution of EEG to approximately 1 cm, and thus developmental changes have been restricted to examinations across the whole brain or across lobes. On the other hand, MEG signals pass through the skull and scalp with little distortion, removing that confound while also allowing for far better spatial resolution of the generating sources of activity, to the order of a few millimetres (Hari and Salmelin, 2012). OPM-MEG specifically offers a significant advantage for studying development compared to traditional MEG systems using superconducting quantum interference devices (SQUIDs; R. M. Hill et al., 2019). Compared to SQUID-MEG, OPM-MEG is more tolerant to head motion, a well-established age-related confound. SQUID-MEG helmets are also “one-size-fits-all”, which means there are coverage, signal strength, and signal-to-noise ratio differences between smaller and larger heads, which is a significant concern for developmental studies. On the other hand, in OPM-MEG the sensors can be mounted on a helmet that is customized to an individual’s head size which can mitigate these issues (Rier et al., 2024).

In this study, we are the first to investigate the development of aperiodic and periodic neural signals using OPM-MEG. We examined age-related changes in the aperiodic slope and offset alongside adjusted peak frequencies and power using a sample of very young children (1 – 5 years of age) and adults (20 – 38 years of age). Leveraging the spatial resolution of MEG, we characterized the developmental changes within individual brain regions, alongside whole-brain analyses.

## 2 Materials and methods

### 2.1 Participants

Seventy-three participants (20 toddlers (1 – 3 years), 16 young children (4 – 5 years), 18 young adults (20 – 26 years), and 19 adults (27 – 38 years)) were recruited as part of a larger study at the Hospital for Sick Children. All participants were typically developing without a current diagnosis or history of a neurological or neurodevelopmental disorder, chromosomal or major congenital abnormality. Individuals were eligible for the current study upon successful completion of the resting-state *Inscapes* paradigm (described in the following section). Written informed consent was provided by the caregiver or participant for the children and adults, respectively, and the study protocol was approved by the Hospital for Sick Children research ethics board.

### 2.2 OPM acquisition

A 40 dual-axis (80 channels) OPM system ((R. M. Hill et al., 2022b); QuSpin Incorporated, Colorado, USA; Cerca Magnetics Limited) with a 1200 Hz sampling rate was used to collect data while participants watched 5-minutes of the *Inscapes* resting-state paradigm (Vanderwal et al., 2015), which consists of a movie of slowly moving shapes and accompanying piano music. Before presenting the paradigm, a 5-minute empty-room noise recording was also obtained. OPM sensors were mounted in one of four possible 3D-printed helmets of varying sizes (Cerca Magnetics Ltd.; three of the four helmets were used in the current study). For each participant, the helmet choice was customized to their head circumference. The participant wore the helmet while seated within a magnetically shielded room (Vacuumschmelze, Hanau, Germany). Bi-planar coil panels and OPM reference sensors (QuSpin Incorporated) were positioned on each side of the participant for dynamic and static nulling of the background magnetic field and its drift, maintaining sensor operation between ±3.5nT (Holmes et al., 2019, Rea et al., 2021). A four-camera system (OptiTrack Flex 13, NaturalPoint Incorporated, Oregon, USA) with infra-red markers placed on the helmet was used to continuously track head movement; head motion data was not successfully acquired for three participants. For co-registration of the OPM data with brain anatomy (R. M. Hill et al., 2020; Zetter et al., 2019), digitisations of the participant’s head with the helmet were acquired using a 3D optical imaging system (Einscan H, SHINING 3D, Hangzhou, China).

### 2.3 Preprocessing

All preprocessing was performed using an OPM preprocessing pipeline developed in-house using the FieldTrip toolbox (version 2202–02–14; (Oostenveld et al., 2011) implemented in MATLAB (version R2021a; (The Mathworks, 2018)). For both the resting-state and empty-room noise recordings, noisy channels were removed using an outlier detection algorithm (Safar et al., 2024), and homogeneous field correction was used to suppress interference from sources outside the head (e.g., environmental noise; R. M. Hill et al., 2022b; Tierney et al., 2021). After bandpass filtering (1–150 Hz, 4th order, two-pass Butterworth), peaks present in the power spectra of both the empty-room and resting-state data were identified as noise and were subsequently band-stop filtered (4th or 3rd order, two-pass Butterworth). The resting-state data were epoched into 1-second segments, and epochs with signals exceeding an artefact rejection threshold were excluded. For each epoch, the maximum head displacement was extracted, and the mean across all epochs was used as an index of head motion for each participant. The common artefact rejection threshold of 4000 fT in MEG studies of young children using SQUIDs (e.g., Alho et al., 2023; W. He et al., 2015; Partanen et al., 2017) was adjusted for the increased noise floor in OPM data using a factor of 13.78 (see Safar et al., (2024) for further details). Participants were included in subsequent analyses if they had at least one minute of resting-state data remaining.

A linear regression identified a significant positive association between age and the number of epochs (*F*(1,63)=17.67, *p*<.001, *β*=0.47) and negative association between age and mean head motion (*F*(1,60)=39.75, *p*<.001, *β*=-0.65), respectively. To ensure these associations did not result in subsequent spurious developmental effects, the number of epochs was matched between the children and adults while minimizing the effect of head motion. For each adult, a number *N* was randomly drawn from the distribution of the number of epochs in the children. Then, the *N* epochs with the highest head motion were selected to be analyzed. Note that given the inherent relation between head motion and age, this effect was still significant after this procedure; however, unlike traditional SQUID-MEG systems, OPMs have been shown to be robust to head motion (Brookes et al., 2022).

The 90 cortical and subcortical regions of the automated anatomical labelling (AAL) atlas (Tzourio-Mazoyer et al., 2002) were reconstructed using a linearly constrained minimum variance (LCMV) beamformer. Forward solutions were computed for each source position using a single-shell head model of an age-appropriate template and assumed a dipole approximation of neural current (Nolte, 2003). For the adults, the International Consortium for Brain Mapping (ICBM) template in standard space (Fonov et al., 2009) was used. For the children, age-specific paediatric templates (Richards et al., 2016) were used, first warping the coordinates of each source in the AAL atlas from standard space to the templates using Advanced Normalization Tools (ANTs; version 2.4.3; Avants et al., 2009). The participants’ head digitisations collected during data acquisition and surface meshes of the age-appropriate templates were used to co-register the head models and source positions to the OPM data (R. M. Hill et al., 2020; Rhodes et al., 2023; Zetter et al., 2019). Covariance matrices for the beamformer were constructed across the continuous data and were regularized using the Tikhonov method with a regularization parameter of 2 % (Tikhonov, 1943). To account for centre of the head biases, the neural activity index was applied by normalizing the beamformer output by the estimated noise (Van Veen et al., 1997).

### 2.4 Power spectra analyses

**Fig. 1:**
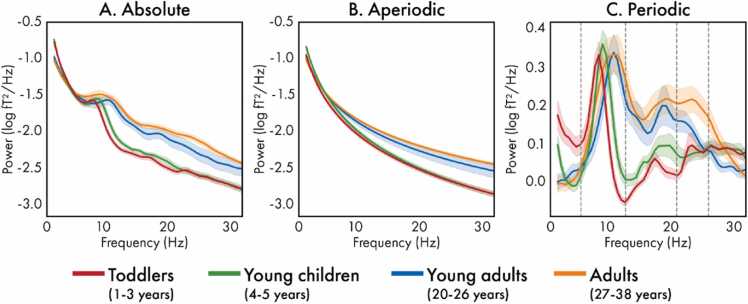
Whole-brain absolute (A), aperiodic (B), and periodic (C) power spectra averaged across the toddlers (1 – 3 years; red), young children (4 – 5 years; green), young adults (20 – 26 years; blue) and adults (27 – 38 years; orange).

The reconstructed timeseries for each brain region were *z*-scored, and the power spectrum density (PSD) was computed using Welch’s method using window length of 1 s with 50 % overlap implemented in MATLAB (version R2021a; (The Mathworks Inc., 2018)); the resulting PSDs were averaged to obtain both whole-brain and regional absolute power spectra. Next, the absolute spectra were parameterized using the SpecParam algorithm (version 1.1.0; formally named Fitting Oscillations and One Over F (FOOOF)) implemented in Python (version 3.11.4), which models the spectra as a combination of aperiodic and periodic components (Donoghue, et al., 2020). The models were fitted between 2 and 40 Hz, without modeling a bend, or knee, in the spectra. The periodic peaks modeled in the spectra were required to have bandwidths at least twice the frequency resolution (0.6 Hz) of the spectra per the developer recommendations, with no other restrictions placed on the number or size of the peaks. The fits of the modelled spectra were visually inspected, and the correlation (*R*-squared) between the raw and fitted spectra and the error of the fitted model was used to evaluate model fit; no outliers (individuals with model fit metrics exceeding three standard deviations from the mean) were identified. For each model, the parameters of the aperiodic component (slope and offset) were extracted; the slope, alternatively called the exponent, captures the steepness of the exponential decay of a power spectrum, while the offset reflects a uniform shift of the spectrum across frequencies (Donoghue et al., 2020). For the periodic component, the peak frequency and corresponding power were identified within bands of interest, selecting the modeled peak with the maximal power if multiple existed. The bands were derived by visually identifying peaks in periodic power spectra (computed by subtracting the aperiodic component from the absolute spectra) averaged across age group (see Fig. 1C in the Results); widths were chosen to ensure the peaks were contained in all age groups. Three bands were identified: alpha (6–12 Hz), low beta (13–20 Hz), and high beta (21–25 Hz).

### 2.5 Statistics

Regressions implemented in MATLAB (version R2021a; (The Mathworks Inc., 2018)) were used to test for associations between age and (a) sex, (b) the data quality measures (number of analyzed epochs, head motion), (c) model fit measures (error and *R*^2^), (d) aperiodic parameters (slope and offset), and (e) periodic parameters (presence or absence of a peak, peak frequency, and power for each frequency band). Logistic and linear regressions were used for binary and continuous variables, respectively, and standardized coefficients were reported as a measure of effect size. For sex, data quality, model fit, and whole-brain aperiodic and periodic measures, significance was held at *p*<0.05. For the regional aperiodic and periodic measures, *p*-values were corrected for multiple comparisons using the false discovery rate (FDR), holding significance at *q*<0.05. Associations between the aperiodic slope and offset were also investigated (see Supplemental information for further information).

There have been numerous demonstrations and discussions about the robustness of OPM-MEG to head motion given its wearable nature (Barry et al., 2019, Boto et al., 2018; R. M. Hill et al., 2019; Pedersen et al., 2022; Seedat et al., 2024; Seymour et al., 2021), and new paradigms have been designed where movement is in fact encouraged (Roberts et al., 2019). Nonetheless, we also demonstrate the lack of association between head motion and the measures of aperiodic and periodic activity in the young adults and adults, for whom age and head motion are not significantly correlated. These findings are presented in the Supplemental information**.**

## 3 Results

### 3.1 Participants

Sixty-nine participants were included in the analysis, after excluding four participants with too few trials remaining after quality control. Participant demographics and data quality measures are summarized by age group (toddlers, children, young adults, and adults) for descriptive purposes in Table 1, alongside regression statistics performed examining associations with continuous age. Age was not significantly associated with sex (*F*(1,67)=0.00, *p*=.977, *β*=0.01). After epoch selection, the number of epochs analyzed was not associated with age (*F*(1,67)=0.32, *p*=.572, *β*=0.07), but mean head motion was still found to significantly decrease with age (*F*(1,64)=33.47, *p*<.001, *β*=-0.60).

**Table 1:** Participant demographics, summarized by age group for descriptive purposes, with regression statistics examining associations with continuous age.

	**Mean [SD]**				**Regression statistics**					
	**Toddlers**	**Young children**	**Young adults**	**Adults**	***F***	***df***	***p*****-value**	***R***^**2**^	***B*****[SE] (×10**^**2**^**)**	***β***
***N***	18	14	18	19	–					
**Age range** **(years)**	1–3	4–5	20–26	27–38	–					
**Age** **(years)**	3.0 [0.8]	5.0 [0.5]	24.0 [1.9]	31.8 [3.5]	–					
**Sex** **(F:M)**	8:10	8:6	11:7	9:10	0.00	(1,67)	.977	0.00	0.05 [1.92]	0.01
**# epochs**	225.2 [68.7]	254.1 [77.6]	221.1 [73.6]	246.6 [59.7]	0.32	(1,67)	.572	0.00	28.01 [66.92]	0.07
**Head motion** **(mm)**	3.0 [2.0]	3.8 [2.4]	1.0 [0.7]	1.0 [0.5]	33.47	(1,64)	<.001	0.34	−9.35 [1.62]	−0.60

^SD: standard deviation; *F*: *F*-statistic; *df*: degrees of freedom; *B*: coefficient; *SE*: standard error; β: standardized coefficient; F: female; M: male^

### 3.2 Power spectra analyses

Whole-brain and regional power spectra were modelled as a combination of aperiodic and periodic components (Donoghue et al., 2020). The whole-brain absolute power spectra are presented in Fig. 1A averaged by age group, alongside the modeled aperiodic power spectra (Fig. 1B) and the periodic power spectra (Fig. 1C). Descriptive statistics for the model fit metrics (*R*^2^ and error) for each age group are presented in Supplemental Table 1; there were no significant associations between age and the whole-brain model fit (*R*^2^: *F*(1,67)=2.16, *p*=.146, *β*=-0.18; error: *F*(1,67)=0.00, *p*=.997, *β*=0.00).

**Fig. 2:**
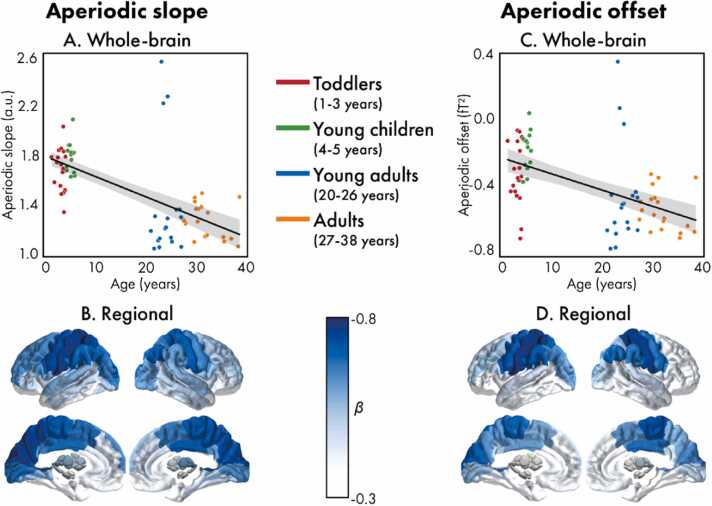
**:** Associations between the aperiodic slope (left: A and B) and offset (right: C and D) for both the whole-brain (top: A and C) and regional (bottom: B and D) power spectra. For the whole-brain spectra, values are coloured according to age group (red: toddlers, green: young children, blue: young adults, orange: adults). For the regional spectra, the standardized coefficients (*β*) for significant (corrected) brain regions are presented.

All statistics for the whole-brain aperiodic and periodic measures are presented in Table 2. The slope of the whole-brain aperiodic component was found to significantly decrease with age (Fig. 2A; *F*(1,63)=40.94, *p*<.001, *β*=-0.62); this association was also significant in all cortical and subcortical brain regions (Fig. 2B), with the strongest effects observed in the left pre- and post-central gyri, the left superior and inferior parietal gyri, and the bilateral cuneus and precuneus. The offset of the whole-brain (Fig. 2C; *F*(1,63)=24.17, *p*<.01, *β*=-0.51) and regional (Fig. 2D) aperiodic components also significantly decreased with age, with the regional effects significant except for the orbital frontal cortices and temporal poles. Decreases in the aperiodic offset followed a similar pattern as the aperiodic slope. Associations between the slope and offset are presented in the Supplemental information.

**Table 2:** Regression statistics examining associations between age and the aperiodic, and periodic parameters.

			**Regression statistics**					
			***F***	***df***	***p*****-value**	***R***^**2**^	***B*****[SE] (×10**^**2**^**)**	***β***
**Aperiodic**	**Slope**		40.94	(1,67)	<.001	0.38	−1.65 [0.26]	−0.62
	**Offset**		24.17	(1,67)	<.001	0.27	−0.99 [0.20]	−0.51
**Periodic**	**Alpha** **(6–12 Hz)**	**Presence of a peak**	–					
		**Frequency**	31.94	(1,67)	<.001	0.32	6.47 [1.14]	0.57
		**Power**	0.01	(1,67)	.943	0.00	0.01 [0.18]	0.01
	**Low beta** **(13–20 Hz)**	**Presence of a peak**	2.07	(1,67)	.155	0.04	7.09 [4.93]	0.90
		**Frequency**	3.82	(1,62)	.055	0.06	−3.91 [2.00]	−0.24
		**Power**	10.95	(1,62)	.002	0.15	0.50 [0.15]	0.39
	**High beta** **(21–25 Hz)**	**Presence of a peak**	2.26	(1,67)	.138	0.04	5.23 [3.48]	0.66
		**Frequency**	1.06	(1,59)	.308	0.02	−1.36 [1.33]	−0.13
		**Power**	9.06	(1,59)	.004	0.13	0.38 [0.13]	0.37

^*F*: *F*-statistic; *df*: degrees of freedom; *B*: coefficient; *SE*: standard error; β: standardized coefficient^

The whole-brain models identified peaks in all participants in the alpha frequency band. In both low and high beta, peaks were identified in 93 % and 88 % of participants, respectively, and the presence of a peak was not associated with age (low beta: *F*(1,67)=2.07, *p*=.155, *β*=0.90; high beta: *F*(1,67)=2.26, *p*=.138, *β*=0.66). The percentage of participants with peaks on a regional level is presented in Supplemental Figure 1; in all frequency bands, there were no significant associations with age in any brain region.

**Fig. 3:**
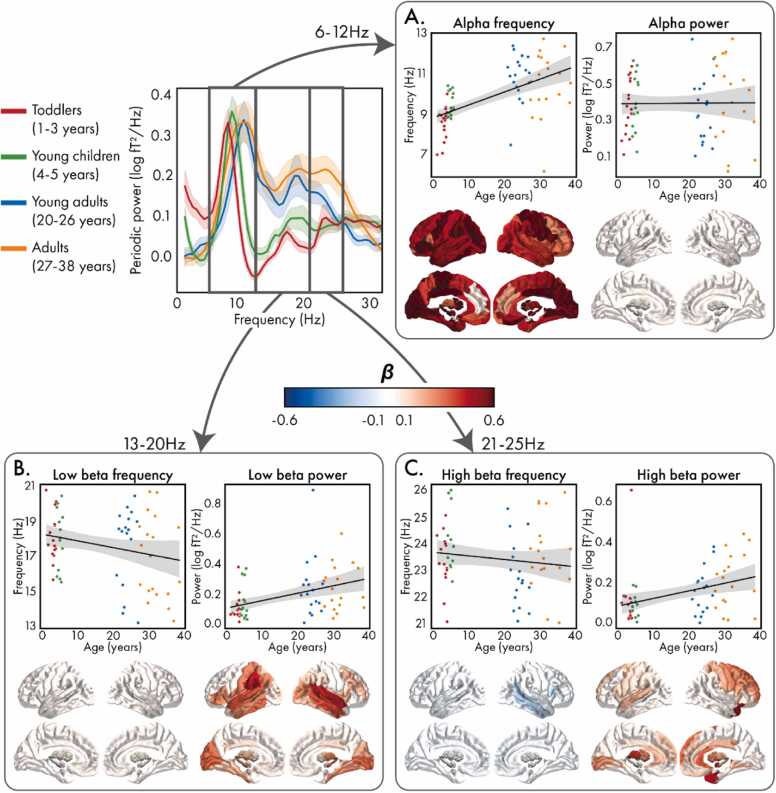
**:** Associations between age and the parameters of the periodic power spectra (center) in the alpha (A), low beta (B), and high beta (C) frequency bands. In each panel, peak frequency (left) and corresponding power (right) are shown for the whole-brain (top) and regional (bottom) power spectra. For the whole-brain measures, values are coloured according to age group (red: toddlers, green: young children, blue: young adults, orange: adults). For the regional spectra, the standardized coefficients (*β*) for significant (corrected) brain regions are presented.

In alpha (6–12 Hz; Fig. 3A), whole-brain peak frequency increased with age (Table 2; *F*(1,67)=31.94, *p*<.001, *β*=0.57), with all brain regions also showing this pattern; largest effects were observed in bilateral parietal regions, particularly the pre- and post-central gyri, medial occipital regions, subcortical regions, and the posterior and median cingulate gyri. Age-related changes in peak alpha power were not significant at a whole-brain (*F*(1,67)=0.01, *p*=.943, *β*=0.01) or regional level. In low beta (13–20 Hz; Fig. 3B), whole-brain peak frequency was not associated with age (*F*(1,62)=3.82, *p*=.055, *β*=0.39), nor in any individual brain region. Low beta power increased with age across the whole brain (*F*(1,62)=10.95, *p*=.002, *β*=0.39) as well as in 74 of the 90 brain regions, with the largest effects occurring in bilateral temporal regions, bilateral occipital regions, particularly the calcarine cortices, and the left supramarginal gyrus. In high beta (21–30 Hz; Fig. 3C), while whole-brain peak frequency did not significantly decrease with age (*F*(1,59)=1.06, *p*=.308, *β*=-0.13), this effect was significant in the opercular part of the right inferior frontal gyrus, the right middle temporal gyrus, and the right Heschl’s gyrus. On the other hand, high beta power increased with age globally (*F*(1,59)=9.06, *p*=.004, *β*=0.37) as well as regionally in 65 regions throughout the brain, with strong effects in the bilateral frontal lobe, particularly in the right hemisphere, the right temporal pole, bilateral subcortical regions, and the anterior cingulate.

## 4 Discussion

This study is the first to use OPMs, a new, wearable MEG technology, to measure brain function in very young children, and the first to examine changes in periodic and aperiodic components of neural signals between very early childhood (1 – 5 years of age) and adulthood (20 – 38 years of age). Consistent with prior EEG studies, we found significant developmental effects of the aperiodic (slope and offset) and periodic (peak frequency and power) features of brain function using OPM-MEG. Additionally, we are the first to show that these changes varied across distinct brain regions. This work not only adds to the growing body of work highlighting the advantages of using OPMs (Pedersen et al., 2022; Rhodes et al., 2023; Safar et al., 2024; Wittevrongel et al., 2021), for a review see (Brookes et al., 2022)), especially for studying development (Boto et al., 2022, Feys and De Tiège, 2024; R. M. Hill et al., 2019), but also contributes novel insights into the spatial pattern of the effect sizes of neurophysiological changes with age.

While EEG and SQUID-MEG can capture the neurophysiological measurements required to fully characterize the development of aperiodic and periodic activity in the brain, they present several important limitations. While EEG is wearable and offers good temporal resolution, the conductive properties of the skull limit its spatial resolution (Hari and Salmelin, 2012), signals are affected by skull thickness (Hoekema et al., 2003), and signals are susceptible to muscle artefacts that occur during head movement (Boto et al., 2018). While SQUID-MEG systems have good temporal and spatial resolution, the need for cryogenic cooling requires the systems to be fixed and have a “one size fits all” helmet, typically designed to fit the average adult head size, which can lead to inhomogeneous coverage and a reduction in signal for smaller compared to larger head sizes; SQUID-MEG signals are also susceptible to head motion (Gross et al., 2013). Given the age-related associations with skull thickness, head size, and head motion, these issues have posed challenges for developmental EEG and SQUID-MEG studies that have spanned early childhood and adulthood (Brookes et al., 2022). OPM-MEG systems address these issues, offering the spatial resolution of SQUID-MEG with the wearable nature of EEG, making it particularly suitable for studying the development of brain function across the life span. Our work is the first to leverage these advantages to examine the development of aperiodic and periodic activity between childhood and adulthood. We show that well-established developmental patterns, such as the increase in peak alpha frequency, can be replicated by this technology, while also providing new insight into brain development that was only possible using OPM-MEG.

### 4.1 Developmental changes in aperiodic signals

We found that both aperiodic slope and offset significantly decreased between toddlerhood and adulthood. By examining changes between early toddlerhood (1 – 5 years of age) and adulthood (20 – 38 years of age), we extend previous developmental EEG work that found similar decreases across smaller age ranges (Cellier et al., 2021, Favaro et al., 2023; A. T. Hill et al., 2022a; McSweeney et al., 2021, McSweeney et al., 2023; Schaworonkow and Voytek, 2021; Tröndle et al., 2022). We also provide important insights into how the effect sizes of these decreases differ across the brain. While previous work has only been able to characterize developmental patterns with broad, lobe-level specificity due to the poorer spatial resolution of EEG (Cellier et al., 2021, Favaro et al., 2023; A. T. Hill et al., 2022a; Schaworonkow and Voytek, 2021), the spatial resolution of OPM-MEG is comparatively much improved, and thus we were able to determine the strength of these changes within individual brain regions.

While the precise neurobiological mechanisms underpinning the aperiodic slope remain unclear, there is evidence to support an association with E/I balance (Gao et al., 2017). Excitatory and inhibitory activity is characterized by faster and slower synaptic currents, respectively, which corresponds to a difference in spectral decay (Gao et al., 2017): increased excitation relative to inhibition results in a flatter power spectrum, while increased inhibition relative to excitation results in a steeper spectrum. The link between E/I balance and the spectral slope has been supported by studies examining changes in the aperiodic slope under pharmacological interventions known to modify inhibitory and excitatory neural activity (Colombo et al., 2019, Gao et al., 2017, Lendner et al., 2020, Medel et al., 2023, Waschke et al., 2021). Furthermore, increases in excitation relative to inhibition can temporally decorrelate spiking in neuronal populations causing neuronal “noise” (Voytek and Knight, 2015), which has been shown to relate to flatter spectral slopes (Pozzorini et al., 2013, Usher et al., 1995). Thus, the observed age-related flattening of the spectral slope may reflect maturational changes in E/I balance, specifically a shift towards excitation with increased age, consistent with the existing developmental EEG work (Cellier et al., 2021, Favaro et al., 2023; A. T. Hill et al., 2022a; McSweeney et al., 2021, McSweeney et al., 2023; Schaworonkow and Voytek, 2021; Tröndle et al., 2022). This conclusion is supported by evidence of protracted changes towards increasingly balanced E/I throughout development and into adulthood that are hypothesized to support plasticity (Larsen et al., 2022, Perica et al., 2022), and maturational increases of neural noise that is thought to reflect the expansion of the brain’s “dynamic repertoire” of functional network configurations (McIntosh et al., 2010).

Shifts in the aperiodic offset can be interpreted as changes in broadband power (Donoghue, Haller, et al., 2020). As such, the aperiodic offset is thought to reflect the overall spiking activity of neuronal populations (Manning et al., 2009, Miller et al., 2009, Miller et al., 2014), and it has been hypothesized that the synaptic pruning, which causes reductions in cortical gray matter throughout childhood, is driving the observed decreases in offset (Cellier et al., 2021, Favaro et al., 2023; A. T. Hill et al., 2022a; McSweeney et al., 2023; Tröndle et al., 2022). However, it has not yet been possible to exclude age-related confounds as the driver of this effect. All published developmental studies to date have used EEG, and increases in skull thickness, which occurs across development, results in decreases in the amplitudes of EEG signals (Hoekema et al., 2003). Given that the aperiodic slope is proportional to broadband power (Donoghue, Haller, et al., 2020), the age-related shifts in the broadband power may simply reflect developmental changes other than the brain (A. T. Hill et al., 2022a; McSweeney et al., 2023). On the other hand, magnetic fields can pass through the skull without distortion (Okada et al., 1999), and thus our OPM-MEG study can conclude that reductions in aperiodic offset with age are genuine. It is well established that cortical gray matter rapidly expands during early childhood, followed by a gray matter reduction in childhood, with protracted changes extending through adolescence (Norbom et al., 2021). The decreases in cortical gray matter can be attributed, in part, to the synaptic pruning and increased myelination that occurs during childhood and adolescence to support the development of efficient neural circuitry (Gogtay et al., 2004, Huttenlocher and Dabholkar, 1997, Paolicelli et al., 2011). Thus, the age-related reductions in global spectral power reported here, and elsewhere (Cellier et al., 2021, Favaro et al., 2023, Gómez et al., 2017; A. T. Hill et al., 2022a; McSweeney et al., 2021, McSweeney et al., 2023; Miskovic et al., 2015; Schaworonkow and Voytek, 2021; Tröndle et al., 2022), which have been shown to parallel the changes in cortical thickness (Whitford et al., 2007), may be due to synaptic pruning and apparent reduction of grey matter.

However, the aperiodic slope and offset are highly correlated (Donoghue et al., 2020). In our study, analyses revealed that the whole-brain aperiodic slope and offset were strongly associated (see the Supplemental information), which is consistent with other examinations of this relation (Favaro et al., 2023, McSweeney et al., 2021, McSweeney et al., 2023, Tröndle et al., 2022). Since we were able to measure the aperiodic components on a regional level, we were also able to demonstrate that there was a high degree of correlation between slope and offset within each brain region, as well as with respect to the patterns of age-related changes across the brain (Supplemental information). While changes in the offset can occur in the absence of a change in the slope (e.g., a vertical shift in the spectrum), a flatter or steeper slope necessitates a co-occurring shift in the offset, if the spectrum is rotated around a non-zero frequency (Donoghue et al., 2020, Ostlund et al., 2022). However, the developmental trajectories of the two components were shown to be distinct in the first months of life across different vigilance stages (Favaro et al., 2023), suggesting that the developmental changes in slope and offset are due to distinct neurophysiological processes (e.g., E/I balance and synaptic pruning, respectively).

The sequence of the development of brain function follows the hierarchical organization of the brain, with the lower-order, sensorimotor cortices maturing by early childhood, while the maturation of higher-order, association cortices is protracted, extending throughout adolescence (e.g., (Dong et al., 2021, Pines et al., 2022), see (Grayson and Fair, 2017, Norbom et al., 2021, Sydnor et al., 2021) for reviews). The pattern of the effect sizes for the age-related changes in the aperiodic components follows a similar pattern: unimodal regions, such as the motor and visual cortices, show larger effects, while higher-order regions, such as the frontal cortices, show smaller effects. This broadly aligns with the existing EEG literature, where studies examining the topography of developmental changes reported steeper decreases in aperiodic activity in parietal-occipital compared to frontal electrodes (Favaro et al., 2023; A. T. Hill et al., 2022a; Schaworonkow and Voytek, 2021). Previous work has provided evidence that aperiodic activity changes non-linearly between early-to-middle childhood, with activity following an inverted-U shaped trajectory, peaking at approximately seven years of age (McSweeney et al., 2023). We propose that our findings coincide with this quadratic developmental model of aperiodic activity and provide evidence that the sequence of development across the brain follows the sensorimotor-association axis. Specifically, if the aperiodic activity of lower-order regions has already peaked, or is close to peaking, during early childhood, changes between childhood and adulthood would be steep. On the other hand, if the development of higher-order regions is protracted, not reaching peak activity until adolescence, changes between childhood and adulthood would be comparatively flatter. While this pattern of non-linear and hierarchical development aligns with the broader functional neuroimaging literature (Hunt et al., 2019, Sydnor et al., 2021), conclusions can not be drawn firmly without examining changes in aperiodic activity across childhood, adolescence, and adulthood.

### 4.2 Developmental changes in periodic signals

Our finding of developmental increases of peak alpha frequency is well-documented (e.g., (Cellier et al., 2021; Cragg et al., 2011; Gómez et al., 2017; A. T. Hill et al., 2022a; Marshall et al., 2002; McSweeney et al., 2023; Miskovic et al., 2015; Rodríguez-Martínez et al., 2017; Tröndle et al., 2022)), and is thought to coincide with increases in the speed of neural communication to facilitate neurocognitive development (Cellier et al., 2021, McSweeney et al., 2023, Segalowitz et al., 2010, Tröndle et al., 2022). This work shows that OPM-MEG replicates this finding and provides insight into the precise spatial patterns of this maturational change. All examined brain regions showed significant increases in peak alpha frequency with age, consistent with a previous report that, aside from increasing frequency with age, the topographies of alpha spectral power were similar between children and adults (Rodríguez-Martínez et al., 2017). We found no changes in alpha power with age aligning with some studies (e.g., Clarke et al., 2001), but not others, with reports of significant age-related increases (e.g., Tröndle et al., 2022) and decreases (e.g., Whitford et al., 2007). A recent, well-powered study reported increases in alpha power between 5 and 22 years of age after adjusting for aperiodic activity and suggested that the conflicting reports were due to the computation of alpha power across canonical frequency bands and unadjusted alpha power (Tröndle et al., 2022). Our methodology was similar to that of Tröndle and colleagues and thus also addressed these concerns, however we were not able to identify an increase in alpha power. While this could be due to different age ranges across the two studies, we also note that different resting-state conditions were used (*Inscapes* in the current study, compared to eyes-closed resting-state). Alpha power in eyes open versus eyes closed resting-states was found to show diverging developmental trajectories (A. T. Hill et al., 2022a; McSweeney et al., 2023), and thus we hypothesize that the lack of reported maturation of alpha power may be due to the use of an alternative, eyes open resting-state paradigm and differing age ranges.

Two peaks were identified in the beta frequency band: low (13 – 20 Hz) and high (21 – 25 Hz). Two beta bands have been identified in resting-state EEG (Rosanova et al., 2009) and MEG (Capilla et al., 2022, Mahjoory et al., 2020) data, and they have been shown to be generated by different mechanisms (Cannon et al., 2014); however, we are one of the first to identify distinct developmental patterns between them. In this study, we found that both beta bands showed increases in power with age. Increased beta power between childhood and adulthood is consistent with previous work (Gómez et al., 2017, Heinrichs-Graham et al., 2018, Schäfer et al., 2014), with evidence to suggest these differences do not emerge until adolescence (A. T. Hill et al., 2022a), and are thought to support the development of sensorimotor and cognitive control (Engel and Fries, 2010). High beta power has also been differentially associated with frontal regions (Capilla et al., 2022, Ferrarelli et al., 2012, Rosanova et al., 2009). For example, transcranial magnetic stimulation (TMS) was found to evoke low beta oscillations in parietal and perirolandic regions, and high beta oscillations in the frontal cortex (Ferrarelli et al., 2012, Rosanova et al., 2009). Relatedly, a data-driven atlas of resting-state oscillations constructed using MEG found that low beta oscillations were specific to lateral occipital-parietal regions, while high beta oscillations were specific to motor and prefrontal cortices (Capilla et al., 2022). This aligns with our finding that age-related changes in high-beta power have the strongest effects in the frontal lobe and is further supported by the increasing changes between the young and slightly older adults, as brain maturation, particularly in the frontal and association cortices continues throughout young adulthood. Interestingly, alongside occipital and parietal regions, temporal regions also showed strong maturational effects of low-beta power. Given the importance of the temporal lobes in social cognition (Olson et al., 2013), this may reflect increases in these skills with age, and warrants further investigation. On the other hand, peak frequency only decreased with age in the high beta band. To our knowledge, only one study from 1999 has investigated the maturational effects of peak frequency separately for low and high beta, and they also found significant decreases in high beta frequency with age, while low beta showed no change (Dustman et al., 1999). Future work is needed to understand the importance of these diverging patterns.

### 4.3 Limitations

This study has several limitations. While we were able to characterize the changes between very young children and adults, our sample lacked participants between 6 and 19 years of age, which is an important period of neurodevelopment. This study leveraged two retrospective OPM-MEG datasets, one of adults which was collected to replicate SQUID-MEG data (Safar et al., 2024), and the other is a sample of typically developing toddlers and young children being collected to study the neurophysiological differences of autism early in life (study is ongoing). OPM-MEG data from school-age children and adolescents were not available, and thus we were unable to include this age range in our analysis. Furthermore, nonlinear maturational trajectories in brain function are prevalent in the neuroimaging literature (Hunt et al., 2019, Sydnor et al., 2021), however, we cannot provide insight into the nature of changes between toddlerhood/early childhood and early adulthood without examining the full spectrum of childhood and adolescence. Our findings must be interpreted in the absence of school-age children and adolescents, and future studies should examine their replicability across development. Our study was also cross-sectional – the generalization of this work to longitudinal changes in the brain is important for future research. While we hypothesize that age-related changes in aperiodic and periodic activity are important for development, future work examining these changes alongside changes in cognition and behaviour will be important for contextualizing these findings. Our findings in deep brain structures should also be interpreted with caution, especially with respect to the measures of periodic activity where effect sizes were large. While the customizable helmets in OPM-MEG addresses the concern of using one-size-fits-all helmets in SQUID-MEG to study development, the smaller head circumferences in children relative to adults means the sensors are relatively closer to subcortical regions in the children compared to adults, which would increase signal-to-noise.

### 4.4 Conclusions

This is the first study to use OPM-MEG to investigate how aperiodic and periodic activity develops in very young children and early adulthood. We found age-related changes in spectral features measured using OPM-MEG that were consistent with the existing literature but leveraged MEG’s spatial resolution to report, for the first time, that the effect sizes of these changes differ throughout the brain. Our work demonstrates the utility of OPM-MEG for studying developmental changes in brain function, especially changes over the early years of life that cannot be reliably measured using traditional adult-sized SQUID-MEG and lays the foundation for future studies to examine aperiodic and periodic activity across the life span and their role in developmental disorders.

## CRediT authorship contribution statement

**Julie Sato:** Writing – review & editing, Project administration, Investigation. **Marlee M. Vandewouw:** Writing – review & editing, Writing – original draft, Visualization, Software, Methodology, Formal analysis, Conceptualization. **Natalie Rhodes:** Writing – review & editing, Investigation. **Kristina Safar:** Writing – review & editing, Investigation. **Margot Taylor:** Writing – review & editing, Writing – original draft, Supervision, Resources, Project administration, Funding acquisition, Conceptualization.

## Declaration of Competing Interest

The authors declare that they have no known competing financial interests or personal relationships that could have appeared to influence the work reported in this paper.

## Data Availability

Due to the novelty of the data and ongoing analysis, raw data will not be made available until a later date.

## References

[bib1] Alho J., Samuelsson J.G., Khan S., Mamashli F., Bharadwaj H., Losh A., McGuiggan N.M., Graham S., Nayal Z., Perrachione T.K., Joseph R.M., Stoodley C.J., Hämäläinen M.S., Kenet T. (2023). Both stronger and weaker cerebro-cerebellar functional connectivity patterns during processing of spoken sentences in autism spectrum disorder. Hum. Brain Mapp..

[bib2] Avants B.B., Tustison N., Song G. (2009). Advanced normalization tools (ANTS). Insight J..

[bib3] Baillet S. (2017). Magnetoencephalography for brain electrophysiology and imaging. Nat. Neurosci..

[bib4] Barry D.N., Tierney T.M., Holmes N., Boto E., Roberts G., Leggett J., Bowtell R., Brookes M.J., Barnes G.R., Maguire E.A. (2019). Imaging the human hippocampus with optically-pumped magnetoencephalography. NeuroImage.

[bib5] Boto E., Holmes N., Leggett J., Roberts G., Shah V., Meyer S.S., Muñoz L.D., Mullinger K.J., Tierney T.M., Bestmann S., Barnes G.R., Bowtell R., Brookes M.J. (2018). Moving magnetoencephalography towards real-world applications with a wearable system. Nature.

[bib6] Boto E., Shah V., Hill R.M., Rhodes N., Osborne J., Doyle C., Holmes N., Rea M., Leggett J., Bowtell R., Brookes M.J. (2022). Triaxial detection of the neuromagnetic field using optically-pumped magnetometry: feasibility and application in children. NeuroImage.

[bib7] Brookes M.J., Leggett J., Rea M., Hill R.M., Holmes N., Boto E., Bowtell R. (2022). Magnetoencephalography with optically pumped magnetometers (OPM-MEG): the next generation of functional neuroimaging. Trends Neurosci..

[bib8] Buzsáki G., Draguhn A. (2004). Neuronal olscillations in cortical networks. Science.

[bib9] Cannon J., Mccarthy M.M., Lee S., Lee J., Börgers C., Whittington M.A., Kopell N. (2014). Neurosystems: brain rhythms and cognitive processing. Eur. J. Neurosci..

[bib10] Capilla A., Arana L., García-Huéscar M., Melcón M., Gross J., Campo P. (2022). The natural frequencies of the resting human brain: an MEG-based atlas. NeuroImage.

[bib11] Cellier D., Riddle J., Petersen I., Hwang K. (2021). The development of theta and alpha neural oscillations from ages 3 to 24 years. Dev. Cogn. Neurosci..

[bib12] Cesnaite E., Steinfath P., Jamshidi Idaji M., Stephani T., Kumral D., Haufe S., Sander C., Hensch T., Hegerl U., Riedel-Heller S., Röhr S., Schroeter M.L., Witte A.V., Villringer A., Nikulin V.V. (2023). Alterations in rhythmic and non-rhythmic resting-state EEG activity and their link to cognition in older age. NeuroImage.

[bib13] Clarke A.R., Barry R.J., McCarthy R., Selikowitz M. (2001). Age and sex effects in the EEG: development of the normal child. Clin. Neurophysiol..

[bib14] Colombo M.A., Napolitani M., Boly M., Gosseries O., Casarotto S., Rosanova M., Brichant J.F., Boveroux P., Rex S., Laureys S., Massimini M., Chieregato A., Sarasso S. (2019). The spectral exponent of the resting EEG indexes the presence of consciousness during unresponsiveness induced by propofol, xenon, and ketamine. NeuroImage.

[bib15] Cragg L., Kovacevic N., McIntosh A.R., Poulsen C., Martinu K., Leonard G., Paus T. (2011). Maturation of EEG power spectra in early adolescence: a longitudinal study. Dev. Sci..

[bib16] Dong H.M., Margulies D.S., Zuo X.N., Holmes A.J. (2021). Shifting gradients of macroscale cortical organization mark the transition from childhood to adolescence. Proc. Natl. Acad. Sci..

[bib17] Donoghue T., Dominguez J., Voytek B. (2020). Electrophysiological frequency band ratio measures conflate periodic and aperiodic neural activity. ENeuro.

[bib18] Donoghue T., Haller M., Peterson E.J., Varma P., Sebastian P., Gao R., Noto T., Lara A.H., Wallis J.D., Knight R.T., Shestyuk A., Voytek B. (2020). Parameterizing neural power spectra into periodic and aperiodic components. Nat. Neurosci*.*.

[bib19] Dustman R.E., Shearer D.E., Emmerson R.Y. (1999). Life-span changes in EEG spectral amplitude, amplitude variability and mean frequency. Clin. Neurophysiol..

[bib20] Ebersole J.S., Pedley T.A. (2003). Current practice of clinical electroencephalography, 3rd edn. Eur. J. Neurol..

[bib21] Engel A.K., Fries P. (2010). Beta-band oscillations — signalling the status quo. Curr. Opin. Neurobiol..

[bib22] Favaro J., Colombo M.A., Mikulan E., Sartori S., Nosadini M., Pelizza M.F., Rosanova M., Sarasso S., Massimini M., Toldo I. (2023). The maturation of aperiodic EEG activity across development reveals a progressive differentiation of wakefulness from sleep. NeuroImage.

[bib23] Ferrarelli F., Sarasso S., Guller Y., Riedner B.A., Peterson M.J., Bellesi M., Massimini M., Postle B.R., Tononi G. (2012). Reduced natural oscillatory frequency of frontal thalamocortical circuits in schizophrenia. Arch. Gen. Psychiatry.

[bib24] Feys O., De Tiège X. (2024). From cryogenic to on-scalp magnetoencephalography for the evaluation of paediatric epilepsy. Dev. Med. Child Neurol..

[bib25] Fonov V.S., Evans A.C., McKinstry R.C., Almli C.R., Collins D.L. (2009). Unbiased nonlinear average age-appropriate brain templates from birth to adulthood. NeuroImage.

[bib26] Gao R., Peterson E.J., Voytek B. (2017). Inferring synaptic excitation/inhibition balance from field potentials. NeuroImage.

[bib27] Gogtay N., Giedd J.N., Lusk L., Hayashi K.M., Greenstein D., Vaituzis A.C., Nugent T.F., Herman D.H., Clasen L.S., Toga A.W., Rapoport J.L., Thompson P.M. (2004). Dynamic mapping of human cortical development during childhood through early adulthood. Proc. Natl. Acad. Sci..

[bib28] Gómez C.M., Rodríguez-Martínez E.I., Fernández A., Maestú F., Poza J., Gómez C. (2017). Absolute power spectral density changes in the magnetoencephalographic activity during the transition from childhood to adulthood. Brain Topogr..

[bib29] Grayson D.S., Fair D.A. (2017). Development of large-scale functional networks from birth to adulthood: a guide to the neuroimaging literature. NeuroImage.

[bib30] Gross J., Baillet S., Barnes G.R., Henson R.N., Hillebrand A., Jensen O., Jerbi K., Litvak V., Maess B., Oostenveld R., Parkkonen L., Taylor J.R., van Wassenhove V., Wibral M., Schoffelen J.M. (2013). Good practice for conducting and reporting MEG research. NeuroImage.

[bib31] Hämäläinen M.S., Lundqvist D. (2019). MEG as an enabling tool in neuroscience: Transcending boundaries with new analysis methods and devices. *Magnetoencephalography: From Signals to Dynamic Cortical*. Netw. Second Ed..

[bib32] Hari R., Salmelin R. (2012). Magnetoencephalography: from SQUIDs to neuroscience: neuroimage 20th anniversary special edition. NeuroImage.

[bib33] He B.J. (2014). Scale-free brain activity: Past, present, and future. Trends Cogn. Sci..

[bib34] He W., Brock J., Johnson B.W. (2015). Face processing in the brains of pre-school aged children measured with MEG. NeuroImage.

[bib35] Heinrichs-Graham E., McDermott T.J., Mills M.S., Wiesman A.I., Wang Y.P., Stephen J.M., Calhoun V.D., Wilson T.W. (2018). The lifespan trajectory of neural oscillatory activity in the motor system. Dev. Cogn. Neurosci..

[bib36] Hill R.M., Boto E., Holmes N., Hartley C., Seedat Z.A., Leggett J., Roberts G., Shah V., Tierney T.M., Woolrich M.W., Stagg C.J., Barnes G.R., Bowtell R.R., Slater R., Brookes M.J. (2019). A tool for functional brain imaging with lifespan compliance. Nat. Commun..

[bib37] Hill R.M., Boto E., Rea M., Holmes N., Leggett J., Coles L.A., Papastavrou M., Everton S.K., Hunt B.A.E., Sims D., Osborne J., Shah V., Bowtell R., Brookes M.J. (2020). Multi-channel whole-head OPM-MEG: Helmet design and a comparison with a conventional system. NeuroImage.

[bib38] Hill A.T., Clark G.M., Bigelow F.J., Lum J.A., Enticott P.G. (2022). Periodic and aperiodic neural activity displays age-dependent changes across early-to-middle childhood. Dev. Cogn. Neurosci..

[bib39] Hill R.M., Devasagayam J., Holmes N., Boto E., Shah V., Osborne J., Safar K., Worcester F., Mariani C., Dawson E., Woolger D., Bowtell R., Taylor M.J., Brookes M.J. (2022). Using OPM-MEG in contrasting magnetic environments. NeuroImage.

[bib40] Hoekema R., Wieneke G.H., Leijten F.S.S., Van Veelen C.W.M., Van Rijen P.C., Huiskamp G.J.M., Ansems J., Van Huffelen A.C. (2003). Measurement of the conductivity of skull, temporarily removed during epilepsy surgery. Brain Topogr..

[bib41] Holmes N., Tierney T.M., Leggett J., Boto E., Mellor S., Roberts G., Hill R.M., Shah V., Barnes G.R., Brookes M.J., Bowtell R. (2019). Balanced, bi-planar magnetic field and field gradient coils for field compensation in wearable magnetoencephalography. Sci. Rep..

[bib42] Hunt B.A.E., Wong S.M., Vandewouw M.M., Brookes M.J., Dunkley B.T., Taylor M.J. (2019). Spatial and spectral trajectories in typical neurodevelopment from childhood to middle age. Netw. Neurosci..

[bib43] Huttenlocher P.R., Dabholkar A.S. (1997). Regional differences in synaptogenesis in human cerebral cortex. J. Comp. Neurol..

[bib44] Larsen B., Cui Z., Adebimpe A., Pines A., Alexander-Bloch A., Bertolero M., Calkins M.E., Gur R.E., Gur R.C., Mahadevan A.S., Moore T.M., Roalf D.R., Seidlitz J., Sydnor V.J., Wolf D.H., Satterthwaite T.D. (2022). A developmental reduction of the excitation:inhibition ratio in association cortex during adolescence. Sci. Adv..

[bib45] Lendner J.D., Helfrich R.F., Mander B.A., Romundstad L., Lin J.J., Walker M.P., Larsson P.G., Knight R.T. (2020). An electrophysiological marker of arousal level in humans. ELife.

[bib46] Mahjoory K., Schoffelen J.M., Keitel A., Gross J. (2020). The frequency gradient of human resting-state brain oscillations follows cortical hierarchies. ELife.

[bib47] Manning J.R., Jacobs J., Fried I., Kahana M.J. (2009). Broadband shifts in local field potential power spectra are correlated with single-neuron spiking in humans. J. Neurosci..

[bib48] Manyukhina V.O., Prokofyev A.O., Galuta I.A., Goiaeva D.E., Obukhova T.S., Schneiderman J.F., Altukhov D.I., Stroganova T.A., Orekhova E.V. (2022). Globally elevated excitation–inhibition ratio in children with autism spectrum disorder and below-average intelligence. Mol. Autism.

[bib49] Marshall P.J., Bar-Haim Y., Fox N.A. (2002). Development of the EEG from 5 months to 4 years of age. Clin. Neurophysiol..

[bib50] McIntosh A.R., Kovacevic N., Lippe S., Garrett D., Grady C., Jirsa V. (2010). The development of a noisy brain. Arch. Ital. De. Biol..

[bib51] McSweeney M., Morales S., Valadez E.A., Buzzell G.A., Fox N.A. (2021). Longitudinal age- and sex-related change in background aperiodic activity during early adolescence. Dev. Cogn. Neurosci..

[bib52] McSweeney M., Morales S., Valadez E.A., Buzzell G.A., Yoder L., Fifer W.P., Pini N., Shuffrey L.C., Elliott A.J., Isler J.R., Fox N.A. (2023). Age-related trends in aperiodic EEG activity and alpha oscillations during early- to middle-childhood. NeuroImage.

[bib53] Medel V., Irani M., Crossley N., Ossandón T., Boncompte G. (2023). Complexity and 1/f slope jointly reflect brain states. Sci. Rep..

[bib54] Merkin A., Sghirripa S., Graetz L., Smith A.E., Hordacre B., Harris R., Pitcher J., Semmler J., Rogasch N.C., Goldsworthy M. (2023). Do age-related differences in aperiodic neural activity explain differences in resting EEG alpha?. Neurobiol. Aging.

[bib55] Miller K.J., Honey C.J., Hermes D., Rao R.P.N., denNijs M., Ojemann J.G. (2014). Broadband changes in the cortical surface potential track activation of functionally diverse neuronal populations. NeuroImage.

[bib56] Miller K.J., Sorensen L.B., Ojemann J.G., Den Nijs M. (2009). Power-law scaling in the brain surface electric potential. PLoS Comput. Biol..

[bib57] Miskovic V., Ma X., Chou C.A., Fan M., Owens M., Sayama H., Gibb B.E. (2015). Developmental changes in spontaneous electrocortical activity and network organization from early to late childhood. NeuroImage.

[bib58] Nolte G. (2003). The magnetic lead field theorem in the quasi-static approximation and its use for magnetoenchephalography forward calculation in realistic volume conductors. Phys. Med. Biol..

[bib59] Norbom L.B., Ferschmann L., Parker N., Agartz I., Andreassen O.A., Paus T., Westlye L.T., Tamnes C.K. (2021). New insights into the dynamic development of the cerebral cortex in childhood and adolescence: Integrating macro- and microstructural MRI findings. Prog. Neurobiol..

[bib60] Okada Y.C., Lahteenmäki A., Xu C. (1999). Experimental analysis of distortion of magnetoencephalography signals by the skull. Clin. Neurophysiol..

[bib61] Olson I.R., McCoy D., Klobusicky E., Ross L.A. (2013). Social cognition and the anterior temporal lobes: a review and theoretical framework. Soc. Cogn. Affect. Neurosci..

[bib62] Oostenveld R., Fries P., Maris E., Schoffelen J.M. (2011). FieldTrip: open source software for advanced analysis of MEG, EEG, and invasive electrophysiological data. Comput. Intell. Neurosci..

[bib63] Ostlund B., Donoghue T., Anaya B., Gunther K.E., Karalunas S.L., Voytek B., Pérez-Edgar K.E. (2022). Spectral parameterization for studying neurodevelopment: how and why. Dev. Cogn. Neurosci..

[bib64] Pani S.M., Saba L., Fraschini M. (2022). Clinical applications of EEG power spectra aperiodic component analysis: a mini-review. Clin. Neurophysiol..

[bib65] Paolicelli R.C., Bolasco G., Pagani F., Maggi L., Scianni M., Panzanelli P., Giustetto M., Ferreira T.A., Guiducci E., Dumas L., Ragozzino D., Gross C.T. (2011). Synaptic pruning by microglia is necessary for normal brain development. Science.

[bib66] Parellada M., Andreu-Bernabeu Á., Burdeus M., San José Cáceres A., Urbiola E., Carpenter L.L., Kraguljac N.V., McDonald W.M., Nemeroff C.B., Rodriguez C.I., Widge A.S., State M.W., Sanders S.J. (2023). In search of biomarkers to guide interventions in autism spectrum disorder: a systematic review. Am. J. Psychiatry.

[bib67] Partanen E., Leminen A., de Paoli S., Bundgaard A., Kingo O.S., Krøjgaard P., Shtyrov Y. (2017). Flexible, rapid and automatic neocortical word form acquisition mechanism in children as revealed by neuromagnetic brain response dynamics. NeuroImage.

[bib68] Pedersen M., Abbott D.F., Jackson G.D. (2022). Wearable OPM-MEG: a changing landscape for epilepsy. Epilepsia.

[bib69] Perica M.I., Calabro F.J., Larsen B., Foran W., Yushmanov V.E., Hetherington H., Tervo-Clemmens B., Moon C.H., Luna B. (2022). Development of frontal GABA and glutamate supports excitation/inhibition balance from adolescence into adulthood. Prog. Neurobiol..

[bib70] Pines A.R., Larsen B., Cui Z., Sydnor V.J., Bertolero M.A., Adebimpe A., Alexander-Bloch A.F., Davatzikos C., Fair D.A., Gur R.C., Gur R.E., Li H., Milham M.P., Moore T.M., Murtha K., Parkes L., Thompson-Schill S.L., Shanmugan S., Shinohara R.T., Satterthwaite T.D. (2022). Dissociable multi-scale patterns of development in personalized brain networks. Nat. Commun..

[bib71] Pozzorini C., Naud R., Mensi S., Gerstner W. (2013). Temporal whitening by power-law adaptation in neocortical neurons. Nat. Neurosci..

[bib72] Rea M., Holmes N., Hill R.M., Boto E., Leggett J., Edwards L.J., Woolger D., Dawson E., Shah V., Osborne J., Bowtell R., Brookes M.J. (2021). Precision magnetic field modelling and control for wearable magnetoencephalography. NeuroImage.

[bib73] Rhodes N., Rea M., Boto E., Rier L., Shah V., Hill R.M., Osborne J., Doyle C., Holmes N., Coleman S.C., Mullinger K., Bowtell R., Brookes M.J. (2023). Measurement of frontal midline theta oscillations using OPM-MEG. NeuroImage.

[bib74] Richards J.E., Sanchez C., Phillips-Meek M., Xie W. (2016). A database of age-appropriate average MRI templates. NeuroImage.

[bib75] Rier L., Rhodes N., Pakenham D.O., Boto E., Holmes N., Hill R.M., Reina Rivero G., Shah V., Doyle C., Osborne J., Bowtell R.W., Taylor M., Brookes M.J. (2024). Tracking the neurodevelopmental trajectory of beta band oscillations with optically pumped magnetometer-based magnetoencephalography. ELife.

[bib76] Roberts G., Holmes N., Alexander N., Boto E., Leggett J., Hill R.M., Shah V., Rea M., Vaughan R., Maguire E.A., Kessler K., Beebe S., Fromhold M., Barnes G.R., Bowtell R., Brookes M.J. (2019). Towards OPM-MEG in a virtual reality environment. NeuroImage.

[bib77] Rodríguez-Martínez E.I., Ruiz-Martínez F.J., Barriga Paulino C.I., Gómez C.M. (2017). Frequency shift in topography of spontaneous brain rhythms from childhood to adulthood. Cogn. Neurodyn.

[bib78] Rosanova M., Casali A., Bellina V., Resta F., Mariotti M., Massimini M. (2009). Natural frequencies of human corticothalamic circuits. J. Neurosci..

[bib79] Safar K., Vandewouw M., Sato J., Devasagayam J., Hill R., Rea M., Brookes M., Taylor M. (2024). Using optically pumped magnetometers to replicate task-related responses in next generation magnetoencephalography. Sci. Rep..

[bib80] Schäfer C.B., Morgan B.R., Ye A.X., Taylor M.J., Doesburg S.M. (2014). Oscillations, networks, and their development: MEG connectivity changes with age. Hum. Brain Mapp..

[bib81] Schaworonkow N., Voytek B. (2021). Longitudinal changes in aperiodic and periodic activity in electrophysiological recordings in the first seven months of life. Dev. Cogn. Neurosci..

[bib82] Seedat Z.A., Pier K.St, Holmes N., Rea M., Al-Hilaly L., Tierney T.M., Embury C.M., Pardington R., Mullinger K.J., Cross J.H., Boto E., Brookes M.J. (2024). Simultaneous whole-head electrophysiological recordings using EEG and OPM-MEG. Imaging Neurosci..

[bib83] Segalowitz S.J., Santesso D.L., Jetha M.K. (2010). Electrophysiological changes during adolescence: a review. Brain Cogn..

[bib84] Seymour R.A., Alexander N., Mellor S., O’Neill G.C., Tierney T.M., Barnes G.R., Maguire E.A. (2021). Using OPMs to measure neural activity in standing, mobile participants. NeuroImage.

[bib85] Sydnor V.J., Larsen B., Bassett D.S., Alexander-Bloch A., Fair D.A., Liston C., Mackey A.P., Milham M.P., Pines A., Roalf D.R., Seidlitz J., Xu T., Raznahan A., Satterthwaite T.D. (2021). Neurodevelopment of the association cortices: patterns, mechanisms, and implications for psychopathology. Neuron.

[bib86] The Mathworks Inc. (2018). MATLAB. In *www.mathworks.com/products/matlab* (2021a).

[bib87] Thuwal K., Banerjee A., Roy D. (2021). Aperiodic and periodic components of ongoing oscillatory brain dynamics link distinct functional aspects of cognition across adult lifespan. ENeuro.

[bib88] Tierney T.M., Alexander N., Mellor S., Holmes N., Seymour R., O’Neill G.C., Maguire E.A., Barnes G.R. (2021). Modelling optically pumped magnetometer interference in MEG as a spatially homogeneous magnetic field. NeuroImage.

[bib89] Tikhonov A.N. (1943).

[bib90] Tröndle M., Popov T., Dziemian S., Langer N. (2022). Decomposing the role of alpha oscillations during brain maturation. ELife.

[bib91] Tzourio-Mazoyer N., Landeau B., Papathanassiou D., Crivello F., Etard O., Delcroix N., Mazoyer B., Joliot M. (2002). Automated anatomical labeling of activations in SPM using a macroscopic anatomical parcellation of the MNI MRI single-subject brain. NeuroImage.

[bib92] Usher M., Stemmler M., Olami Z. (1995). Dynamic pattern formation leads to noise in neural populations. Phys. Rev. Lett..

[bib93] Van Veen B.B.D., Van Drongelen W., Yuchtman M., Suzuki A. (1997). Localization of brain electrical activity via linearly constrained minimum variance spatial filtering. IEEE Trans. Biomed. Eng..

[bib94] Vanderwal T., Kelly C., Eilbott J., Mayes L.C., Castellanos F.X. (2015). Inscapes: a movie paradigm to improve compliance in functional magnetic resonance imaging. NeuroImage.

[bib95] Voytek B., Knight R.T. (2015). Dynamic network communication as a unifying neural basis for cognition, development, aging, and disease. Biol. Psychiatry.

[bib96] Voytek B., Kramer M.A., Case J., Lepage K.Q., Tempesta Z.R., Knight R.T., Gazzaley A. (2015). Age-related changes in 1/f neural electrophysiological noise. J. Neurosci..

[bib97] Waschke L., Donoghue T., Fiedler L., Smith S., Garrett D.D., Voytek B., Obleser J. (2021). Modality-specific tracking of attention and sensory statistics in the human electrophysiological spectral exponent. ELife.

[bib98] Whitford T.J., Rennie C.J., Grieve S.M., Clark C.R., Gordon E., Williams L.M. (2007). Brain maturation in adolescence: Concurrent changes in neuroanatomy and neurophysiology. Hum. Brain Mapp..

[bib99] Wittevrongel B., Holmes N., Boto E., Hill R., Rea M., Libert A., Khachatryan E., Van Hulle M.M., Bowtell R., Brookes M.J. (2021). Practical real-time MEG-based neural interfacing with optically pumped magnetometers. BMC Biol..

[bib100] Zetter R., Iivanainen J., Parkkonen L. (2019). Optical Co-registration of MRI and On-scalp MEG. Sci. Rep..

