# Supplementary material for: The development of aperiodic and periodic resting-state power between early childhood and adulthood: New insights from optically pumped magnetometers
Source: Dev Cogn Neurosci. 2024 Aug 10;69:101433. doi: 10.1016/j.dcn.2024.101433 (PMC11350249; doi:10.1016/j.dcn.2024.101433)
Supplement: Supplementary file 1 — Supplementary material. [file mmc1.docx]

# Supplemental information

## Model fit metrics

**Supplemental Table 1**: Model fit metrics, summarized by age group for descriptive purposes, with regression statistics examining associations with continuous age.

|  | **Mean [SD]** | | | | **Regression statistics** | | | | | |
| --- | --- | --- | --- | --- | --- | --- | --- | --- | --- | --- |
|  | **Toddlers** | **Young children** | **Young adults** | **Adults** | ***F*** | ***df*** | ***p*-value** | ***R*^2^** | ***B* [SE]**  **(×10^2^)** | ***β*** |
| ***R*^2^** | 0.995 [0.005] | 0.998 [0.001] | 0.996 [0.002] | 0.995 [0.003] | 2.16 | (1,67) | .146 | 0.03 | -0.00 [0.00] | -0.18 |
| **Error** | 0.023 [0.011] | 0.016 [0.005] | 0.019  [0.076] | 0.021 [0.007] | 0.00 | (1,67) | .997 | 0.00 | -0.00 [0.01] | 0.00 |

^SD: standard deviation;^ *^F^*^:^ *^F^*^-statistic;^ *^df^*^: degrees of freedom;^ *^B^*^: coefficient;^ *^SE^*^: standard error; β: standardized coefficient^

## Regional presence of peaks


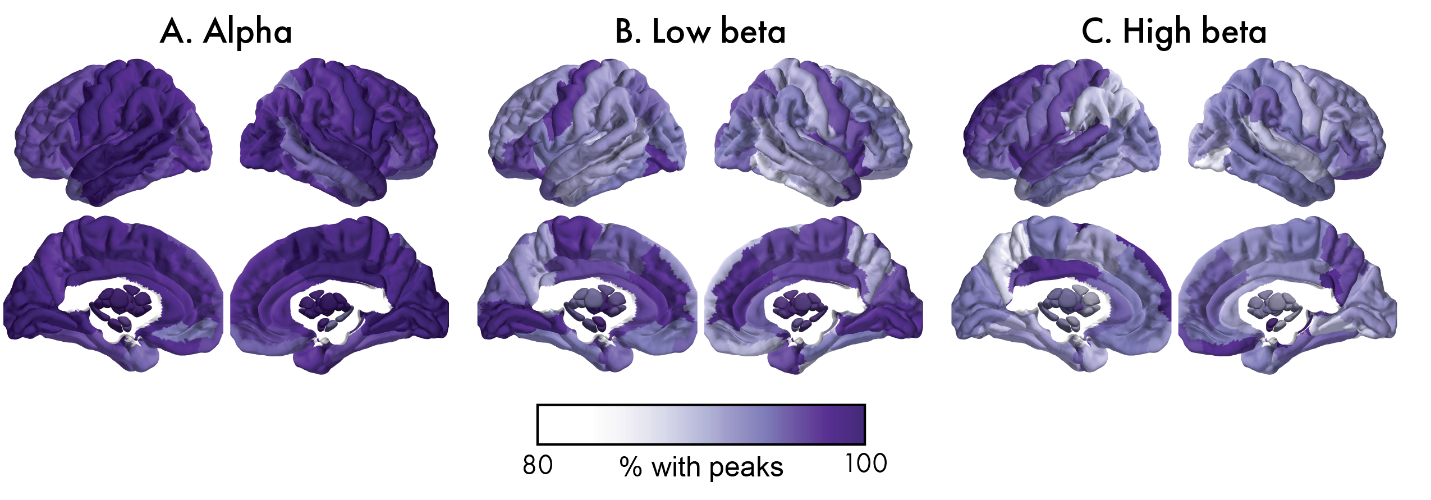


**Supplemental Figure 1**: Percent of participants with detectable peaks for each brain region for alpha (A), low beta (B), and high beta (C).

## Associations between aperiodic slope and offset

To examine the association between whole-brain aperiodic slope and offset, we performed a linear mixed effects regression with offset as the dependent variable, slope as the independent variable, and participant-specific random intercepts. A significant positive association was found (*F*(1,67)=443.3, *p*<.001, *β*=0.93). We also applied these models to each brain region, FDR-correcting for multiple comparisons. Significant positive associations were observed in each of the 90 brain regions, with effect sizes (standardized coefficients, equivalent to Pearson correlation coefficients for these models) from 0.75 to 0.95. To examine whether age-related changes in the aperiodic slope and offset were also associated across the brain, a regression was performed between the regional effect sizes (**Figure 2B** and **2D**). Again, a significant association was found (*F*(1,88)=805.1, *p*<.001, *β*=0.95).

## Associations with head motion

Within the young adult and adult participants, there was no significant association between head motion and age (*F*(1,33)=0.16, *p*=.694, *β*=-0.08). In this sample, there were no significant associations between head motion and the whole-brain aperiodic and periodic features (**Supplemental Table 2**), nor were there any significant associations at the regional level. On top of the established robustness of OPM-MEG to head motion, the lack of association between head motion and aperiodic/periodic activity in the adult samples buttresses our conclusions that our findings are not being driven by age-related differences in head motion.

**Supplemental Table 2**: Regression statistics examining associations between head motion and the aperiodic, and periodic parameters in the young adult and adult samples, which show no association between head motion and age.

|  |  | | **Regression statistics** | | | | | |
| --- | --- | --- | --- | --- | --- | --- | --- | --- |
|  |  | | ***F*** | ***df*** | ***p*-value** | ***R*^2^** | ***B* [SE] (×10^2^)** | ***β*** |
| **Aperiodic** | **Slope** | | 1.33 | (1, 35) | .257 | 0.04 | -1.34 [1.16] | -0.19 |
|  | **Offset** | | 1.10 | (1, 35) | .301 | 0.03 | -0.84 [0.80] | -0.17 |
| **Periodic** | **Alpha**  **(6-12Hz)** | **Presence of a peak** |  | | | | | |
|  |  | **Frequency** | 0.06 | (1, 35) | .803 | 0.00 | -1.22 [4.84] | -0.04 |
|  |  | **Power** | 0.00 | (1, 35) | .950 | 0.00 | 0.04 [0.72] | 0.01 |
|  | **Low beta**  **(13-20Hz)** | **Presence of a peak** | 0.05 | (1, 35) | .830 | 0.00 | 4.92 [22.69] | 0.24 |
|  |  | **Frequency** | 1.82 | (1, 34) | .186 | 0.05 | -10.66 [7.90] | -0.22 |
|  |  | **Power** | 0.15 | (1, 34) | .693 | 0.00 | 0.25 [0.62] | 0.07 |
|  | **High beta**  **(21-25Hz)** | **Presence of a peak** | 0.11 | (1, 35) | .738 | 0.00 | -4.89 [14.51] | -0.24 |
|  |  | **Frequency** | 0.80 | (1, 33) | .376 | 0.02 | 4.26 [4.76] | 0.15 |
|  |  | **Power** | 4.10 | (1, 33) | .051 | 0.11 | 0.84 [0.42] | 0.33 |

*^F^*^:^ *^F^*^-statistic;^ *^df^*^: degrees of freedom;^ *^B^*^: coefficient;^ *^SE^*^: standard error; β: standardized coefficient^
